# Recurrent Syncope in Patients with Carotid Sinus Hypersensitivity

**DOI:** 10.5402/2012/216206

**Published:** 2012-09-10

**Authors:** Alfonso Lagi, Sergio Cerisano, Simone Cencetti

**Affiliations:** ^1^UO Emergency Medicine and Syncope Unit, Ospedale Santa Maria Nuova-Firenze, Piazza S. Maria Nuova, 1-50131, Florence, Italy; ^2^Cardiology Unit, Electrophysiology and Syncope Unit, Ospedale Santa Maria Nuova-Firenze, Piazza S. Maria Nuova, 1-50131, Florence, Italy

## Abstract

Syncope recurrence in pacemaker-implanted subjects for the cardio-inhibitory response to sinus carotid massage (SCM) was investigated. The study-hypothesis was that recurrences had significant vasodepressor responses that could justify the loss of consciousness. Forty-six patients were enrolled (16 patients and 30 controls), followed and revaluated after 5–7 years. At the end of follow-up, significant differences were found between patients and controls in mean SCM SAP (87 *versus* 106 mmHg) and reduction in mean SCM SAP (59 *versus* 38 mmHg); in the number of symptomatic subjects soon after SCM (5 *versus* 1); and in the number of subjects suffering from orthostatic hypotension. A subgroup of 13 patients showed significantly different hypotensive responses to SCM compared with the values observed at study recruitment. The data showed that some subjects with a defined hemodynamic pattern in response to SCM may change their characteristics and have spontaneous and/or provocative symptoms. These data explain the syncopal relapses, and suggest the presence of autonomic dysregulation in individuals with carotid sinus hypersensitivity.

## 1. Introduction

Carotid sinus hypersensitivity (CSH) is the response manifested with bradycardia and/or hypotension during sinus carotid massage (SCM). CSH has been classified as “cardio-inhibitory” (asystole >3 s), “vasodepressive” (if systolic arterial pressure (SAP) falls to >50 mmHg), or “mixed” forms [1]. The effect of SCM may be not only upon cardiovascular parameters but also on symptoms. Hence, the appearance of symptoms during SCM and their characteristics are the most important signs for appropriate and efficacious therapy. 

Attention has recently been focused upon differences between CSH and carotid sinus syndrome (CSS). CSS is diagnosed in the presence of symptoms, syncope, or presyncope, during or soon after SCM [2]. Dual-chamber pacing (DDD) is the treatment of choice for cardioinhibitory and mixed forms. It is believed to improve quality of life by reducing the number of episodes of recurrent syncope, but it does not completely eliminate the risk of syncope recurrence [3], especially in patients with vasodepressive effects [4, 5]. 

It is known that patients with CSH showing a reduction in blood pressure after an SCM have a worse prognosis than those with a pure cardioinhibitory response or a lack of vasodepressor response [1, 4]. Hence, the aim of this study was to assess if patients treated with cardiac pacing for cardio-inhibitory CSH with recurrent syncope had a prevalent vasodepressor response that could justify loss of consciousness. 

## 2. Methods

The study protocol was approved by the Ethics Committee of Santa Maria Hospital (Firenze, Italy). All subjects gave written informed consent to be involved in the study.

All subjects who had had pacemaker implantation for cardio-inhibitory CSH were followed up. They were recruited at Santa Maria Hospital from 1 January 2002 to 31 December 2004. They were checked for presence/absence of recurrent syncope (>1) and split in two subgroups: patients with >1 episode of syncope and controls without syncope or 1 syncope only. They were clinically evaluated and a new SCM done to record the response. Each subject had two SCMs, the first at enrolment and pacemaker implantation and the last at reevaluation. 

### 2.1. Patients

All subjects were studied for the response to SCM: cardiovascular parameters (heart rate and SAP), symptoms (syncope or presyncope) and comorbidities were evaluated. Reduction in SAP was considered to be an absolute value and as a change from baseline after SCM (SCM SAP). 

The enrolled patients met the criteria shown in Table 1.

All participants were asked to refrain from smoking and from eating heavy meals on the day of CSM (only breakfast was allowed).

Patients were classified according to the criteria shown in Table 2.

Patients were excluded if they: (i) had postcritical symptoms (drowsiness or mental confusion) and/or loss of consciousness lasting >20 min as assessed from the patient's description (if credible) or from a witness or (ii) discontinued ongoing hypotensive therapy. 

### 2.2. Procedure

Patients and controls were subjected to SCM at 60° on a head-up tilt table 5 days after stopping the use of drugs that affect blood pressure; heart rate and blood pressure were recorded beat by beat. Pressure of ≥5 s and ≤10 s was applied in a bilateral sequential fashion beginning with the right side and, if the results were negative, continuing to the left side [3–5]. The procedure was stopped if symptoms or diagnostic hemodynamic parameters were seen. 

In the first evaluation and in the statistical analysis, only SAP values were taken into account because they are considered more reliable. The lower SAP value after the end of the SCM and at ≤30 s was chosen for statistical analyses [6]. 

Afterwards, patients were classified based on effective or ineffective responses at SCM. They were distinguished between the decrease in SAP more (effective massage) or less than 50 mmHg (ineffective massage) and as the difference from the baseline value [2, 7]. Data were transferred to an analysis program to obtain adequate identifying evidence. The symptoms associated with SCM were syncope (loss of consciousness), presyncope (episode of near fainting), and asymptomatic (any unspecified sensation considered to be nondiagnostic).

The SAP values at baseline and at SCM as well as the related symptoms and the presence/absence of orthostatic hypotension (OH) at physical examination between the two subgroups were evaluated. Afterwards, the results of effective SCM only between patients and controls were compared. The data compared were baseline SAP; SCM SAP; the difference between SCM SAP at enrolment and when subjects were implanted with a pacemaker; SCM SAP at the reevaluation. 

## 3. Statistical Analyses

Baseline demographics and hemodynamic indices were compared between groups using an analysis of variance for continuous data and the *χ* ^2^ test for categorical data. Comparisons were made between patients and controls. *P* < 0.05 was considered significant.

## 4. Results

Fifty-five subjects were recruited for pacemaker implantation. The mean age was 73 ± 6 years (range, 65–88 years). The female : male ratio was 1.16. They were evaluated after 5–7 years. In this period, there were 7 deaths, and 2 patients were lost to followp. Finally, the study was done in 46 subjects, 16 with >1 episode of syncope/presyncope (34% of subjects), and 30 without or one episode of syncope only. The mean followup was 72 months. The characteristics of the patients and controls are shown in Table 3. 

There were no significant differences between the two subgroups apart from an increase in the intake of angiotensin-converting enzyme inhibitors (ACEi). The differences in the number of all episodes of syncope and syncope/year (which were the inclusion or exclusion criteria for the subgroups) appeared to be considerably different, more than eightfold those seen in subgroups of patients. Furthermore, episodes of presyncope were much more frequent in patients (about tenfold more). However, the most significant difference was in the prevalence of OH (31% *versus *10%) and the total number of injuries (12 times *versus* 1).

The results of SCM are represented in Table 4. In the two subgroups of 16 patients and 30 controls, the reduction of SCM SAP had significant difference (*P* < 0.01) between patients and controls (87 *versus* 106 mmHg) and the mean reduction of SCM SAP was 59 *versus* 38 mmHg (*P* < 0.05), respectively. At last 5 patients had syncope (symptomatic) during SCM compared with 1 in the controls. Five patients had OH at the end of standing compared with 3 in the controls.

Table 5 shows the results for subjects who had an effective SCM: they were 13 patients (10 on the right side and 3 on the left side) and 5 controls. The mean difference between the baseline SAP and SCM SAP was 73 *versus* 62 mmHg (*P* = ns). There was no significant difference between the SCM SAP at enrolment (100 *versus* 90 mmHg) and at reevaluation (68 *versus* 83 mmHg) in patients compared with controls, respectively. There was a significant difference between the mean SCM SAP at enrolment, 5 years before, and the mean SCM SAP at the reevaluation (100 *versus* 68 mmHg (*P* < 0.05) and 90 *versus* 83 mmHg (*P* = ns) in patients and controls, respectively.

## 5. Discussion

Sixteen patients (35%) were selected for evaluation of recurrence of episodes of syncope on followup compared with those seen in controls. The two study populations appeared to be comparable with respect to the major variables (Table 3). The differences were in the total number of episodes and for the number of episodes of syncope/presyncope per year, as well as for falls which resulted in trauma. Furthermore, a significant difference in the prevalence of OH was characterized for the two populations. 

The aim of this work was to ascertain if patients had significant differences in the prevalence of vasodepressive responses to SCM, that is, events that could be related to syncope recurrence. The results were in accordance with this hypothesis. Patients had a vasodepressive response that was significantly different from that seen in controls (Table 4) because the mean SCM SAP was lower than the mean fall in SAP from the baseline SAP (59 *versus *38 mmHg). Cases with symptoms and those with OH were significantly different in patients compared with controls (*P* < 0.01). 

We must focus on two areas: the number of patients with recurrence of syncope and those with a vasodepressive response to SCM. Syncope recurrence was seen in 35% of subjects. A recent review report stated that the percentage of cases suffering from CSS is 0–20% at five-year followup [8]. Hence, in the present study, the number of subjects with recurrent syncope appeared to be high. 

The hypotensive response to SCM is rarely estimated if the subject is lying down. In the present study, 45% subjects (patients and controls together) had a hypotensive response to SCM. The hypotensive response to SCM is very frequent in older populations and is associated with the cardio-inhibitory form in 60–87% of cases [9–12]. Hence, the subjects in the present study represented a specific population. That is, at the beginning of recruitment they were homogeneous with respect to symptoms and cardio-inhibitory response to SCM; at the end of followup their characteristics had changed because they had syncope recurrence and a different response to SCM. 

Furthermore, in a subgroup among the patients (13 cases) and controls (5 cases) who had an effective response to SCM, the patients had a SCM SAP less than that observed 5 years previously (68 *versus* 100 mmHg). The five controls had the same response (Table 5). 

The aim of the present study seems to have been confirmed because the patients selected and treated for cardio-inhibition changed their patterns at SCM as defined by a new vasodepressive response, by symptoms recurrence, and by the appearance of OH. Finally, when 46 subjects were recruited for SCM cardio-inhibition, 35% had recurrent syncope, 28% had a vasodepressive effective SCM, and from these, 10% had symptoms during the maneuver. 

This was the first study to show that some patients can modify their responses not only hemodynamically but also symptomatically, thereby witnessing a translation from CSH to CSS [2]. 

Dysautonomia may occur in these patients, thereby confirming the results of studies which underscore a link between CSH and OH [13] or suggest the presence of autonomic dysregulation in individuals with CSH, indicating that CSH is a generalized autonomic disorder [14]. It may be presumed that such vasodepression is present in subjects with cardio-inhibitory CSH who have not benefited from pacemaker implantation [15]. 

## 6. Conclusion

These data showed that some subjects with a defined hemodynamic pattern in response to SCM may change their characteristics and have spontaneous and/or provocative symptoms. These data explain the syncopal relapses and suggest the presence of autonomic dysregulation in individuals with carotid sinus hypersensitivity.

## Figures and Tables

**Table 1:** Criteria for study inclusion.

DDD carriers	
Age > 65 years	
Absence of coronary acute syndrome (defined as a rise in levels of cardiac troponin with supportive evidence in the form of typical symptoms and/or electrocardiographic changes)	
Absence of acute disease at enrolment and at reevaluation	
Absence of use of hypotensive drugs or their suspension for ≥5 days before CSM	

**Table 2:** Criteria for patient classification.

Number of episodes of syncope and/or presyncope per patient/year occurring after pacemaker implantation	
Trauma (defined as emergency department access)	
Drug history	
Clinical examination	
Blood test (glycemia, creatinine level, hematocrit)	
Telemetry monitoring for ≤48 h to exclude dangerous arrhythmias	
Presence of orthostatic hypotension detected after the first 1 min and after 3 min while standing up and defined as a change of ≥20 mmHg in systolic arterial pressure and 10 mmHg in diastolic arterial pressure	
Response to SCM carried out in the upright position as hypotension and/or symptoms	

**Table 3:** Studied patients' and controls' characteristics.

	Patients	Controls	*P*
Number	16	30	
Males	11	12	
Mean age	71 (6)	72 (5)	Ns
Diabetes mellitus	3	5	Ns
Hypertension	8	12	Ns
Coronary heart disease	5	9	Ns
Heart failure	1	2	Ns
Renal failure	1	3	Ns
Atrial fibrillation	2	4	Ns
Stroke/TIA	4	5	Ns
CODP	2	4	Ns
Neoplasia	1	2	Ns
LDL-cholesterol mg/dl	112	131	Ns
ACEi/ARB	14	21	0.04
*β* blockers	6	12	Ns
CC blockers	5	11	Ns
Diuretics	4	11	Ns
*α* blockers	2	4	Ns
OH	5 (31%)	3 (10%)	<0.01
no. episodes of syncope	43	5	<0.01
no. episodes of presyncope	102	11	<0.01
Syncope and presyncope/ year from PM implant	1.3	0.16	<0.01
Injury	12	1	<0.01

**Table 4:** Baseline and after sinus carotid massage characteristics of the two subgroups.

	Baseline SAP	Mean SCM SAP	Mean fall SAP	Symptomatic subjects	OH
Patients no. 16	147 (±14)	87 (±31)	59 (±26)	5 (31%)	5 (31%)
Controls no. 30	144 (±13)	106 (±19)	38 (±13)	1 (3%)	3 (10%)
*P*	ns	<0.01	<0.05	*P* < 0.01	*P* < 0.01

SAP: systolic arterial pressure in mmHg.

OH: orthostatic hypotension.

**Table 5:** Subject with effective massage: Difference for SAP after SCM.

	Mean SCM SAP difference from baseline SAP	Mean SCM SAP enrollment	Mean SCM SAP revaluation	*P*
Patients n° 13	73 (±12)	100 (±15)	68 (±10)	<0.05
Controls n° 5	62 (±4)	90 (±14)	83 (±9)	ns
*P*	ns	ns	ns	

SCM SAP: systolic arterial pressure in mmHg after sinus carotid massage.

Mean SCM SAP difference from baseline indicates the mean fall of SAP after the sinus massage from the baseline SAP at the time of enrollment and the implantation of PM.

Mean SCM SAP enrollment indicates the mean value of SAP after sinus massage.

Mean SCM SAP Revaluation indicates the mean value of SAP at the end of the followup.

0.05 indicates the difference from Mean SCM SAP Enrollment and Mean SCM SAP Revaluation.
